# Improved Quantification of Cerebral Vein Oxygenation Using Partial Volume Correction

**DOI:** 10.3389/fnins.2017.00089

**Published:** 2017-02-27

**Authors:** Phillip G. D. Ward, Audrey P. Fan, Parnesh Raniga, David G. Barnes, David L. Dowe, Amanda C. L. Ng, Gary F. Egan

**Affiliations:** ^1^Monash Biomedical Imaging, Monash UniversityClayton, VIC, Australia; ^2^Faculty of Information Technology, Monash UniversityClayton, VIC, Australia; ^3^Department of Radiology, Lucas Center for Imaging, Stanford UniversityStanford, CA, USA; ^4^The Australian eHealth Research Centre, CSIRO Health and BiosecurityHerston, QLD, Australia; ^5^Monash eResearch Centre, Monash UniversityClayton, VIC, Australia; ^6^Department of Anatomy and Neuroscience, The University of MelbourneMelbourne, VIC, Australia; ^7^ARC Centre of Excellence for Integrative Brain FunctionMelbourne, VIC, Australia

**Keywords:** partial volume, vein, oxygen extraction fraction, OEF, quantitative susceptibility mapping, QSM, MRI

## Abstract

**Purpose:** Quantitative susceptibility mapping (QSM) enables cerebral venous characterization and physiological measurements, such as oxygen extraction fraction (OEF). The exquisite sensitivity of QSM to deoxygenated blood makes it possible to image small veins; however partial volume effects must be addressed for accurate quantification. We present a new method, Iterative Cylindrical Fitting (ICF), to estimate voxel-based partial volume effects for susceptibility maps and use it to improve OEF quantification of small veins with diameters between 1.5 and 4 voxels.

**Materials and Methods:** Simulated QSM maps were generated to assess the performance of the ICF method over a range of vein geometries with varying echo times and noise levels. The ICF method was also applied to *in vivo* human brain data to assess the feasibility and behavior of OEF measurements compared to the maximum intensity voxel (MIV) method.

**Results:** Improved quantification of OEF measurements was achieved for vessels with contrast to noise greater than 3.0 and vein radii greater than 0.75 voxels. The ICF method produced improved quantitative accuracy of OEF measurement compared to the MIV approach (mean OEF error 7.7 vs. 12.4%). The ICF method provided estimates of vein radius (mean error <27%) and partial volume maps (root mean-squared error <13%). *In vivo* results demonstrated consistent estimates of OEF along vein segments.

**Conclusion:** OEF quantification in small veins (1.5–4 voxels in diameter) had lower error when using partial volume estimates from the ICF method.

## Introduction

Important physiological information including venous oxygenation can be quantified from MRI scans in a minimally invasive manner. Venous oxygen extraction fraction (OEF) can be derived from quantitative susceptibility mapping (QSM) (Salomir et al., 2003; Marques and Bowtell, 2005) by examining the magnetic susceptibility difference between veins and reference tissue [e.g., cerebrospinal fluid (CSF)]. OEF measurements provide critical information for diagnosis and prognosis in many neurological disorders, including multiple sclerosis and stroke (Baron et al., 1981; Fan et al., 2015). However, partial volume effects lead to errors in OEF estimation from vessels of small diameter.

Analysis of OEF and other physiological measures from MRI scans requires delineation of regions of interest (ROIs), in the form of binary masks, to identify which voxels represent the structures of interest. The non-regular geometry of biological structures (e.g., vessels) and the regular grid of MRI are fundamentally mismatched, which manifests as partial volume effects at boundaries. Interestingly, in some cases small biological structures such as veins are only visible in MR gradient echo magnitude images due to partial volume which causes signal cancelation between parenchyma and venous blood (Reichenbach et al., 2000).

An intuitive approach to handling partial volume errors in the analysis of large structures is to morphologically erode an ROI, such that the remaining central voxels have minimal or no partial volume effects. Following this erosion a mean or median operation can be applied, however, for veins smaller than three voxels in diameter the erosion operation may leave few (if any) voxels in the mask. The mean of such a small sample is highly sensitive to noise, and approaches the maximum voxel intensity as the sample size decreases. Without the erosion operation, the partial volume effects are likely to cause systematic underestimation by a mean or median approach.

Other approaches to perfect partial volume correction (PPC) include fitting Gaussian mixture models (Shattuck et al., 2001; Tohka et al., 2004; Brouwer et al., 2010) with separate classes for single component voxels and for voxels containing partial volume effects. A linear model can then be applied to the partial volume voxels to estimate their components. Neighborhood or spatial information has also been included to identify voxel components (Manjón et al., 2010). These approaches require a sufficient number of non-partial volume voxels with minimal noise to fit the components of the models and are in general not suitable for cerebral veins with small radii.

Instead, an established approach is to use the maximum intensity voxel (MIV) to represent the vessel (Fan et al., 2014). This method discards potentially useful voxels and is highly sensitive to noise. After performing a MIV operation important morphological characteristics, such as spatial gradients, are ill defined or impossible to calculate.

Promising vein-specific approaches based on modeling magnetic moments in gradient echo MRI have been recently proposed to estimate the magnetic susceptibility, orientation and size of veins (Hsieh et al., 2015a,b; McDaniel et al., 2016). These methods directly model the MR gradient echo signal, which is particularly sensitive to vein orientation and echo time (Li et al., 2012). Interpreting this signal requires a complicated model to discern the underlying structures (veins) from local intravascular signal and non-local extravascular effects. As such, these approaches can be highly sensitive to orientation and require orientation-specific acquisition parameters, or alternatively be limited to specific vein orientations.

Instead of directly modeling MR gradient echo signals, we used QSM techniques to calculate the magnetic susceptibility in veins. The resulting voxel intensities depict local magnetic susceptibility with negligible orientation or echo time dependencies (Fan et al., 2014; Sood et al., 2016). However, these dependencies are substantially smaller than those of gradient echo magnitude and/or phase images (Li et al., 2012, 2014; Fan et al., 2014; Wang and Liu, 2015).

The objectives of this paper are to introduce a novel cylindrical geometry estimation method called Iterative Cylindrical Fitting (ICF) and to validate its use for correcting partial volume errors in small veins. The ICF method resolves the position and radius of small cylindrical structures, to construct partial volume maps, using the voxel intensities of cross-sectional image slices. The partial volume maps enable improved estimation of the true intensities of tubular structures such as veins by using all available voxels, including boundary voxels. The ICF method reduces the effect of noise in the measurements by increasing the number of data points used. In this paper the ICF method is applied to small veins in QSM maps to improve OEF measurements. However, the technique can also be applied to any linear partial volume problem for cylindrical geometries.

## Materials and methods

### ICF method

The ICF technique takes two inputs, an image (in this case a QSM map) and an initial binary vein mask, and outputs the vein geometry, vein magnetic susceptibility, and a partial volume map for each cross-sectional slice. The initial vein mask may be obtained through manual delineation or by an automated method (Ward et al., 2015) and is dilated to include adjacent voxels that may not contain pure vein signal.

Cross-sectional slices of the Cartesian image grid, in which a vein appears circular or elliptical, are processed individually. Slices are cropped to contain the vein and immediate neighborhood (in practice 4–10 voxels are included around the vein in all directions). Analysis of each slice provides an estimate of vein radius, $\hat{R}$, center position, $\hat{P}=\left({\hat{p}}_{x},\;{\hat{p}}_{y}\right)$, and vein magnetic susceptibility, ${\hat{\chi }}_{\text{vein}}$. For clarity, estimates are denoted with hats, e.g., ${\hat{\chi }}_{\text{vein}}$ is an estimate of the true quantity χ_vein_. The ICF method assumes that the susceptibility value (measured voxel intensity in a QSM map), χ_*i*_, of the voxel, *i*, is a linear combination (Shattuck et al., 2001; Tohka et al., 2004; Manjón et al., 2010) of the vein intensity and the surrounding tissue intensity, χ_background_, weighted by the partial volume fraction, ρ_*i*_, which ranges from zero to one:

(1) $$\begin{array}{r} \chi_{i}=\rho_{i}\;\cdot \chi_{\text{vein}}+\left(1-\rho_{i}\right)\cdot \chi_{\text{background}} \end{array}$$

The ICF method is not specific to QSM, and the use of χ is for clarity in this particular application. In the general case, χ could be replaced with *S* to refer to signal intensity in the input image.

ICF has the following iterative pattern and uses an initial estimate of χ_background_ from an ROI outside of the dilated binary vein mask:

1. The initial partial volume values, ${\hat{\rho }}_{i}$, are estimated from the dilated vein mask, i.e., ${\hat{\rho }}_{i}=1$ inside the dilated vein mask and ${\hat{\rho }}_{i}=0$ for voxels outside.
2. A “vein-only image” is produced by subtracting the background component of the image, ${\hat{\chi }}_{\text{vein}-\text{only},\text{ i}}=\chi_{i}-{\hat{\chi }}_{\text{background}}\left(1-{\hat{\rho }}_{i}\right)$.
3. The vein geometry ($\hat{P},\;\hat{R}$) is estimated from ${\hat{\chi }}_{\text{vein}-\text{only},\text{ i}}$ using (Equations 3–8) (see Appendix A in Supplementary Material for derivation).
4. A new set of non-binary ${\hat{\rho }}_{i}$ values is calculated from the vein geometry.
5. Steps 2–4 are then repeated until a convergence criterion is met, e.g., a minimal change in ${\hat{\chi }}_{\text{vein}-\text{only},\text{ i}}$, or a termination condition reached, e.g., a maximum number of iterations.

**Figure 1:**
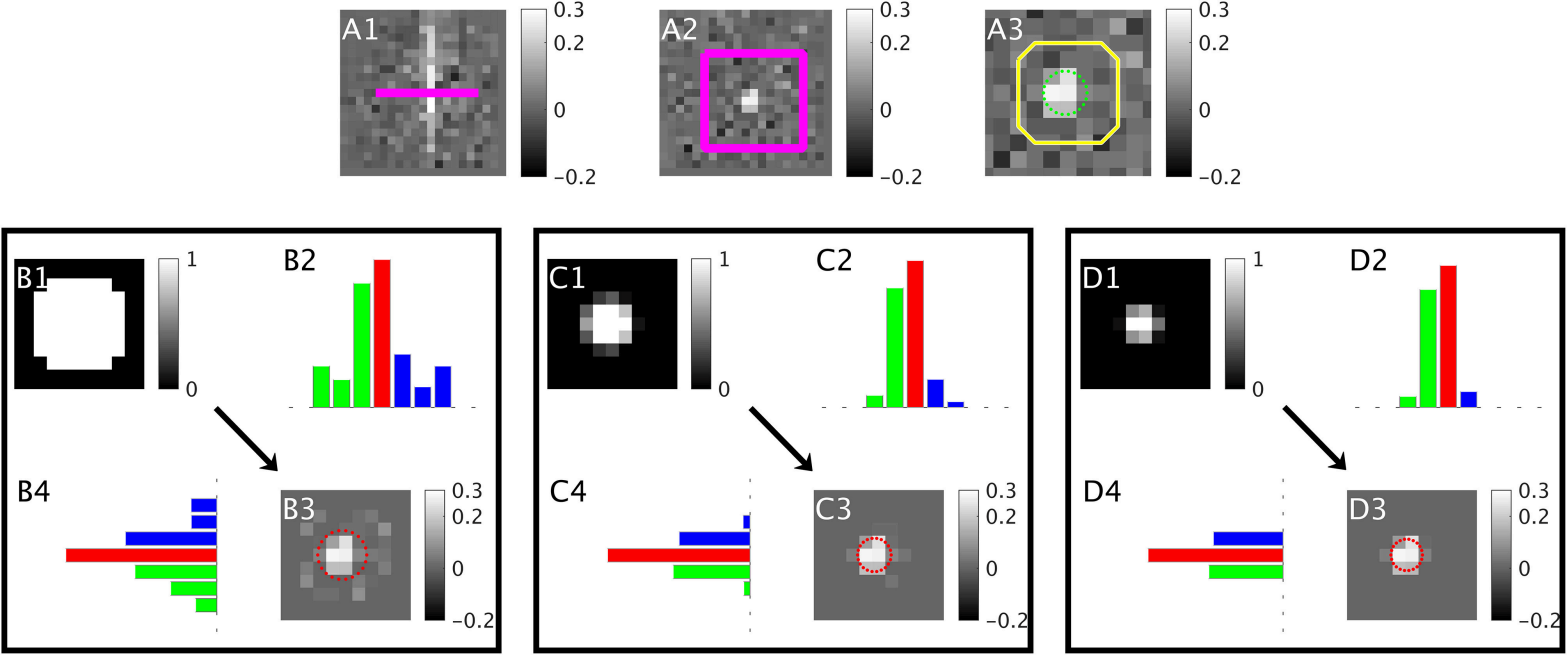
**A depiction of the iterative ICF method over the first three iterations. (A)** Input QSM in parallel **(A1)** and a cross sectional slice **(A2)** with the slice location and neighborhood overlaid in pink. **(A3)** shows a close-up of the neighborhood region with the true vein boundary in green and a dilated vein mask in yellow. ${\hat{\chi }}_{\text{background}}$ is estimated from outside the dilated mask. **(B1)** shows the initial partial volume values taken from the dilated vein mask. **(B3)** is the “vein-only image” produced by subtracting the background component from the input QSM **(A3)** using the partial volume map. **(B2,B4)** are the column and row summations respectively of **(B3)**, red shows the centermost column (highest cumulative intensity), green and blue correspond to the segment areas used to estimate the vein geometry. The geometry estimated from **(B3)** is overlaid in red. This geometry is used to provide the partial volume in the next iteration **(C1)**. The process is repeated in **(C,D)**.

This process is depicted in Figure 1. After the iterative process finishes, ${\hat{\chi }}_{\text{vein}}$ can be calculated from all voxels within the cross-sectional image slice:

(2) $${\hat{\chi }}_{\text{vein}}=\arg{\text{ min}}_{{\hat{\chi }}_{\text{vein}}}\sum_{i}{\left(\left({\hat{\chi }}_{\text{vein}}\cdot {\hat{\rho }}_{i}\text{​}-\text{​}{\hat{\chi }}_{\text{background}}\cdot \left(1\text{​}-\text{​}{\hat{\rho }}_{i}\right)\right)\text{​}-\text{​}\chi_{i}\right)}^{2}$$

**Figure 2:**
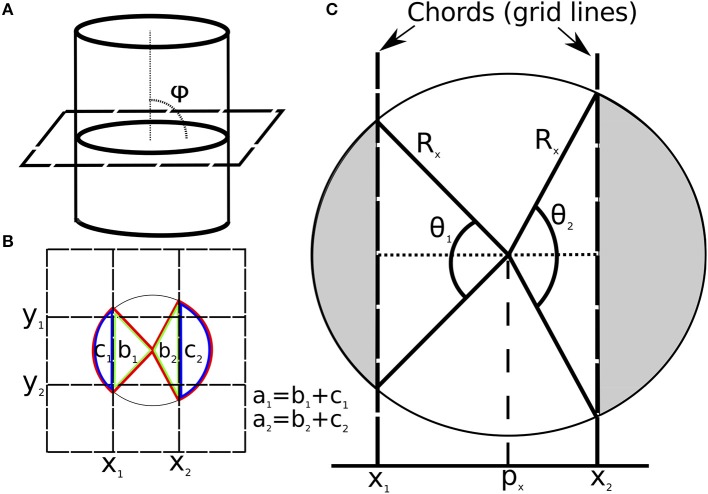
**(A)** Diagram showing the angle (φ = 90°) between an image slice and a vein. **(B)** Vein cross-section overlaid with image grid. Grid lines are selected as chords for the x-axis (*x*_1_, *x*_2_) and y-axis (*y*_1_, *y*_2_). The sector for *x*_1_ and *x*_2_ are labeled (in red) *a*_1_ and *a*_2_ respectively. The triangles for *x*_1_ and *x*_2_ are labeled (in green) *b*_1_ and *b*_2_ respectively. The segment area for *x*_1_ and *x*_2_ are labeled (in blue) *c*_1_ and *c*_2_ respectively. **(C)** Diagram of vein cross-section on image plane overlaid with model parameters for the x-axis. The shaded sections show the circular area outside the image grid lines (segments). Note: ICF is not limited to the case of φ = 90°, this is chosen to simplify the diagram.

To calculate $\hat{P}$, $\hat{R}$ and ${\hat{\rho }}_{i}$ (Step 3), ICF assumes that the vein appears as an ellipse on the cross-sectional image slice (Figure 2A). $\hat{P}$ and $\hat{R}$ are calculated along each image axis separately. The procedure for the x-axis (columns) is detailed here, and is the same for the y-axis (rows). For each cross-sectional slice (Figure 2A), the voxel/pixel boundaries (image grid lines) of the centermost column in the image are identified (*x*_1_ and *x*_2_ in Figure 2B). The centermost column is defined as the column with the highest cumulative image intensity within the cropped image (red columns in Figure 1).

The section of a grid line (*x*_1_ or *x*_2_) that intersects the elliptical disk is a chord. The chord delineates a region of the disk defined as a segment (*c*_1_ for *x*_1_ and *c*_2_ for *x*_2_ in Figure 2B). The area of this disk segment (*c*_1_ or *c*_2_) is proportional to the sum of voxel intensities (here, magnetic susceptibility) in each segment (green and blue columns in Figure 1). This area is divided by the total intensity in the cropped image to give the segment area (*A*_*k*_) as a fraction of the vein cross-sectional area.

(3) $$\begin{array}{r} A_{k}=\frac{\sum_{i}^{M}{\hat{\chi }}_{\text{vein}-\text{only},\text{i}}}{\sum_{j}^{N}{\hat{\chi }}_{\text{vein}-\text{only},\text{j}}} \end{array}$$

where *j* denotes all the voxels in the cropped image slice (*N*), and *i* denotes all voxels in columns preceding grid line *x*_1_ for *k* = 1 (and succeeding grid line *x*_2_ for *k* = 2). The voxels (*M*) indexed by the latter subscript (*i*) are depicted in Figure 2B as the first and last columns (for *k* = 1 and *k* = 2 respectively), and as the green and blue columns in Figures 1B–D parts 2 and 4. These two segment areas (shown in Figure 2B as *c*_1_ and *c*_2_) are used to calculate the angles, θ_1_ and θ_2_ (Figure 2C) using (Equation 4).

(4) $$\begin{array}{r} A_{k}=\frac{1}{2\pi }\left(\theta_{k}-\sin\theta_{k}\right) \end{array}$$

Although Equation (4) does not have an analytic form for θ_*k*_ in terms of *A*_*k*_, in practice an iterative solver provides a highly accurate approximation of θ_*k*_ for a given value of *A*_*k*_. The ellipse radii (*R*_*x*_ and *R*_*y*_) and center (*p*_*x*_, *p*_*y*_) can then be calculated using (Equations 5 and 6).

(5) $$\begin{array}{r} p_{x}=x_{1}+\left(x_{2}-x_{1}\right)\frac{\cos\frac{\theta_{1}}{2}}{\cos\frac{\theta_{1}}{2}+\cos\frac{\theta_{2}}{2}} \end{array}$$

(6) $$\begin{array}{r} R_{x}=\;\frac{x_{2}-x_{1}}{\cos\frac{\theta_{1}}{2}+\cos\frac{\theta_{2}}{2}} \end{array}$$

Equations for *p*_*y*_ and *R*_*y*_ are identical to Equations (5) and (6) with θ_1_ and θ_2_ calculated using *A*_*k*_ from rows of the image rather than columns.

The vein radius (*R*) can then be calculated from either *R*_*x*_ or *R*_*y*_ using the vein tilt angle, φ, relative to the x-y plane (Equations 20 and 21). In this work, the radius of the vein was determined as the average of *R*_*x*_ and *R*_*y*_ according to Equations (7).

(7) $$\begin{array}{r} R=\frac{1}{2}\left(\frac{R_{x}}{\sqrt{{\left(\frac{\sin\phi }{\cos\varphi }\right)}^{2}+\cos^{2}\phi }}+\frac{R_{y}}{\sqrt{{\left(\frac{\cos\phi }{\cos\varphi }\right)}^{2}+\sin^{2}\phi }}\right) \end{array}$$

The tilt angle (φ in Figure 2A) can be calculated by fitting a line to (*p*_*x*_, *p*_*y*_) across adjacent slices using the slices above and below the slice of interest. Alternatively, or for initialization, the tilt angle may be extracted from 2nd order partial derivatives of the image (Frangi et al., 1998).

### Oxygen extraction fraction (OEF)

OEF measurements are made using the magnetic susceptibility difference between venous voxels and CSF. In this study we use a uniform hematocrit (Hct) value of 0.4 (Fan et al., 2014).

(8) $$\begin{array}{r} OEF=\frac{\left(\chi_{\text{vein}}-\chi_{\text{background}}\right)}{\chi_{do}\cdot Hct} \end{array}$$

The difference in magnetic susceptibility between fully deoxygenated hemoglobin and oxygenated hemoglobin (χ_*do*_ = 4π · 0.27ppm) was taken from the literature (Spees et al., 2001).

In numerical simulations χ_background_ was the mean of the voxels outside of a dilated vein mask. For *in vivo* images, χ_background_ was estimated from the mean susceptibility in ventricular CSF, as recent studies have found reduced inter-subject variance by using CSF as a reference region compared to other regions (Straub et al., 2016).

### Numerical simulations

Numerical synthetic QSM maps were used to assess systematic error and the performance characteristics of ICF with different vein geometries, magnetic field orientations, noise characteristics and sequence parameters. A description of all default simulation parameters used can be found in Table 1 including the ranges for those that varied for all QSM maps, such as vein orientation and center position.

**Table 1:** **Default values and ranges for synthetic dataset parameters**.

**Parameter**	**Fixed value/Range**
Vein position relative to voxel center (in 2 transverse axes)	[−0.5, 0.5]^*^ voxels
Vein radius (“apparent vein radius” in final image)	1.3 voxels
Vein radius (input vein radius prior to truncation)	8 voxels
Vein orientation relative to image plane (Θ)	[−45°, 45°]^*^
Vein orientation relative to image x-axis (ϕ)	[0°, 45°]^*^
Magnetic susceptibility contrast (χ_vein_−χ_background_)	0.30 ppm
Field strength	7 tesla
Transverse decay constant for vein (Deistung et al., 2008)	7.4 ms
Transverse decay constant for background (Deistung et al., 2008)	33.2 ms
Longitudinal decay constant for vein (Deistung et al., 2008)	2,587 ms
Longitudinal decay constant for background (Deistung et al., 2008)	2,132 ms
Proton density for vein (Deistung et al., 2008)	0.90
Proton density for background (Deistung et al., 2008)	0.77
Repetition time (TR)	25 ms
Echo time (TE)	7.65 ms
Gaussian noise (standard deviation, std. noise)	0.1

Ranges (^*^) were sampled randomly with a uniform distribution for all synthetic datasets.

**Figure 3:**
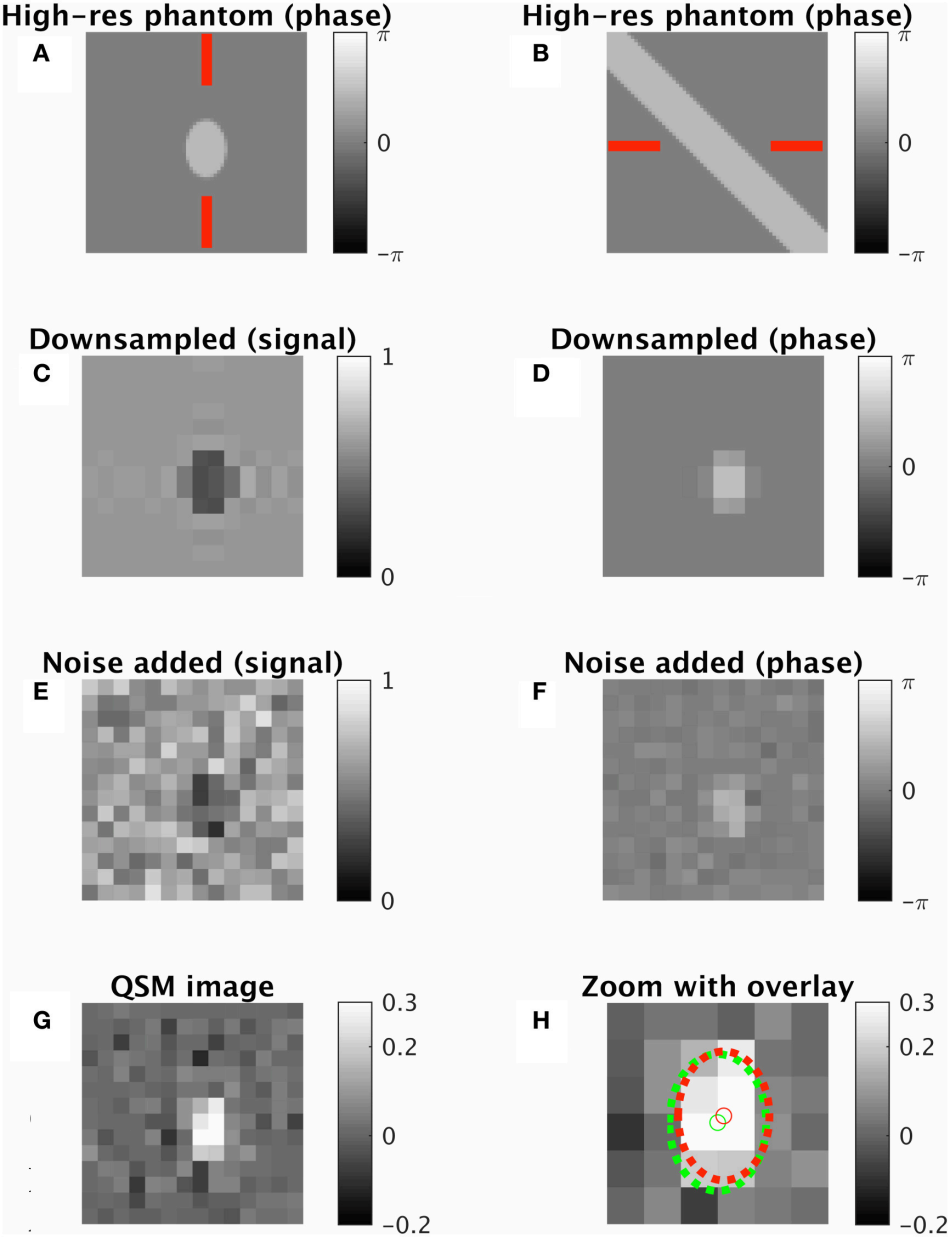
**An example of the synthetic data generation using default parameters from Table 1**. A single cross-sectional slice is shown of a vein oriented 45° away from the imaging plane (radius 1.3 voxel widths, cross-sectional area approximately 7.5 squared voxel widths). **(A,B)** High-resolution phase image two orthogonal views (shown by red lines). **(C,D)** Downsampled image with magnitude image **(C)** and phase image **(D)**. **(E,F)** Downsampled complex image with noise added. **(G)** QSM map taken from processed image. **(H)** Zoomed QSM map overlaid with vein center and perimeter for ground truth (green) and ICF prediction (red). True χ_vein_ = 0.3 ppm. ICF estimate 0.35 ppm. MIV estimate 0.42 ppm. ICF radius estimate 1.17 voxels.

For a given set of parameters, a perfect cylinder of infinite length was modeled with a radius of 8 voxels. This “ideal case” model was sampled onto a discrete matrix by taking the complex sum of 200 randomly sampled points per voxel. The complex gradient recalled echo signal at each point was simulated with longitudinal and transverse decay (Deistung et al., 2008), and phase from the local magnetic field using an infinite cylinder model (Sedlacik et al., 2011). The signal matrix (128 × 128 × 128) contained isotropic voxels. Different experiments for different vein radius to voxel size ratios were performed by truncating the matrix in k-space (downsampling) to the required vein radius to voxel size ratio. Gaussian noise was added to the real and imaginary components separately to give a Rician distribution in the downsampled complex image (Gudbjartsson and Patz, 1995). An example of this process, with intermediate images, is depicted in Figure 3.

QSM maps were reconstructed using iterative least-squares regression (iLSQR) from the STI-Suite (Wu et al., 2012; Li et al., 2014). The QSM maps were normalized so the mean of the region outside a dilated vein mask had the same background value as χ_background_.

The three experiments explored the effects of echo time, noise, and vein radius. The ranges explored can be found in Table 2. The three experiments were simulated twice, once with the magnetic field oriented parallel to the vein axis, and once with a perpendicular orientation. In total, six sets of experimental results were collected. For each experiment 300 images were generated, each with a random vein position and image axis orientation, to examine different elliptical cross sections and asymmetric partial volume profiles.

**Table 2:** **Experimental variations in default data simulation parameters**.

**Experiment**	**Variation**	**Range**
1	Echo time (TE)	[3, 24] ms
2	Gaussian noise (standard deviation, std. noise)	[0.05, 0.5]
3	Vein radius (“apparent vein radius” due to image resolution)	[0.56, 2.1] voxels

The phase images used to compute the QSM maps contained noise, truncation artifacts, and partial volume effects, along with signal-to-noise profiles influenced by signal decay and decoherent phase. These effects are all influenced by varying vein orientation, vein position, elliptical cross-sections, and vein radius/image resolution. Note that resolution and radius are coupled as the apparent vein radius was controlled by k-space truncation. Experimentally the degree of downsampling varied from approximately 16:1 to 4:1 depending on the required apparent vein radius in different experimental conditions.

The contrast-to-noise ratio (CNR) of each simulation was approximated as the QSM difference between background and vein divided by noise. The noise was estimated by the standard deviation of the QSM map outside of the dilated vein mask. Fit error (ϵ_fit_) was defined as the mean square of the residuals across voxels (*N*) that contained (or were estimated to contain) vein signal ($\rho_{i}+{\hat{\rho }}_{i}>0$).

(9) $$\begin{array}{r} \epsilon_{fit}=\frac{1}{N}\overset{N}{\underset{i}{\sum }}{\left(\chi_{i}-\left({\hat{\chi }}_{\text{vein}}\cdot {\hat{\rho }}_{i}+{\hat{\chi }}_{\text{background}}\cdot \left(1-{\hat{\rho }}_{i}\right)\right)\right)}^{2} \end{array}$$

ICF was performed using the middle three adjacent slices of the simulated phantom using a vein mask from known geometry dilated by three voxels. The convergence criterion was a change in ϵ_fit_ of less than 0.001. A maximum 15 iterations was also applied as a termination condition. The radius and center were averaged across slices using inverse-error weights ($\epsilon_{\text{fit}}^{-1}$), and then ${\hat{\chi }}_{\text{vein}}$ was estimated for the middle slice.

Three analysis methods were employed alongside the ICF method. The first method attempted to mitigate partial volume by taking the MIV from the centermost slice. The second method incorporated no partial volume correction (NPC) by calculating the mean of all voxels with non-zero partial volume. Finally, an ideal method of PPC with ground truth partial volume maps (i.e., ${\hat{\rho }}_{\text{PPC}}=\rho$) was applied to solve Equation (1). As such, PPC examined the error attributed to the linear partial volume assumption (Equation 1) and QSM reconstruction.

OEF values were derived according to Equation (8) using the vein magnetic susceptibility estimates from all four methods (ICF, MIV, NPC, and PPC). The error was calculated (ϵ_*v*_) between these four estimates and the true OEF value. The ICF geometry estimates were also compared with the true values [position (ϵ_*P*_), radius (ϵ_*R*_), and partial volume maps (ϵ_ρ_)]. Partial volume error was calculated using all voxels with non-zero partial volume in either the true partial volume map or the estimated map, i.e., $N=\left\{i:\rho_{i}>0\;\vee \;{\hat{\rho }}_{i}>0\right\}$.

(10) $$\begin{array}{r} \epsilon_{v}=\hat{OEF}-OEF \end{array}$$

(11) $$\begin{array}{r} \epsilon_{P}=\sqrt{{\left({\hat{p}}_{x}-p_{x}\right)}^{2}+{\left({\hat{p}}_{y}-p_{y}\right)}^{2}} \end{array}$$

(12) $$\begin{array}{r} \epsilon_{R}=\frac{\left|\hat{R}-R\right|}{R} \end{array}$$

(13) $$\begin{array}{r} \epsilon_{\rho }=\sqrt{\frac{1}{N}\overset{N}{\underset{i}{\sum }}{\left({\hat{\rho }}_{i}-\rho_{i}\right)}^{2}} \end{array}$$

Errors were reported as the mean absolute value over a set of images ± the standard error. The median and inter-quartile range of ϵ_*v*_ for all four methods, both magnetic field orientations (parallel and perpendicular) and three sets of experimental parameter ranges were plotted for comparison.

### Systematic error investigation

Numerical simulations of specific vein center positions, zero added noise and a higher resolution matrix were used to examine error from the QSM reconstruction process and the rasterization of the gradient-recalled echo signal.

The complex gradient-recalled echo signal was simulated on a 512 × 512 × 512 matrix with a vein radius of 32 voxels. The matrix was then downsampled by a factor of 24.6 to yield an apparent vein radius of 1.3 voxels. Default experimental parameters were used for all other values (Table 1). QSM images were then computed from the downsampled image using the same process as described in Section Numerical Simulations.

Three vein center positions were simulated, the corner of a voxel, the center of a voxel, and half way between these two positions. Both parallel and perpendicular magnetic fields were applied, resulting in six simulated images that explored asymmetric partial volume profiles and field orientations.

The PPC, ICF, and MIV methods were performed upon this set of images and compared.

### *In vivo* data

All *in vivo* data was acquired with consent from The University of Melbourne Human Research Ethics Committee. The subject provided informed signed consent prior to taking part in the study. Thirty vein segments were analyzed from the healthy volunteer who was scanned using a 7T research MRI scanner (Siemens, Erlangen, Germany) with a 32-channel head and neck coil. The protocol was a multi-echo gradient-recalled echo sequence (TE = [7.65 ms, 11.5 ms, 15.3 ms], TR = 18 ms, voxel = 0.6 mm isotropic, matrix = 316 × 366 × 224, flip angle = 13°, GRAPPA factor 3). The first echo was fully flow compensated and used for QSM processing. STI-Suite (Wu et al., 2012; Li et al., 2014) was used to remove phase wraps, background phase, and reconstruct the QSM maps.

The vein segments were manually identified and each cross-sectional slice was analyzed. ICF was performed in two passes, the first assuming the vein was perpendicular to the slice, and the second using a vein orientation calculated from a linear fit of the vein positions calculated in the first pass. The convergence and termination criteria were the same as for the synthetic data. The mean radius across all slices was used to estimate ${\hat{\chi }}_{\text{vein}}$ for each slice. Experiments were performed in MATLAB (Version r2015b) on a dual Intel X5650 (12M Cache, 2.66 GHz, 6.40 GT/s Intel QPI) computer. MIV estimates were also made of each *in vivo* vein for comparison.

## Results

### Numerical simulation results

Geometry estimates were examined across all simulations. The mean partial volume map error (ϵ_ρ_) was 12.9%±0.3%, the mean vein position error (ϵ_*P*_) was 0.33±0.01 voxels, and the mean vein radius error (ϵ_*R*_) was 26.9% ± 1.0%.

The mean absolute error of OEF measurements (ϵ_*v*_) for all four methods across the six simulated conditions can be found in Table 3. The PPC method that uses known geometry to solve Equation (1) showed the lowest error in all cases (5.48% averaged across the conditions). The average error across all six conditions was 7.7% for the ICF approach, compared to 12.4% and 14.4% for the MIV and NPC approaches respectively. The ICF approach had significantly less error than the MIV and NPC approaches when compared across all simulated images (Wilcoxon signed-rank test *p* < 10^−5^).

**Table 3:** **Mean absolute-error in OEF estimates (ϵ_*v*_) for the ICF, MIV, NPC and PPC methods for each experiment and field orientation**.

**Experiment**	**Orientation of magnetic field**	**ICF**	**MIV**	**NPC**	**PPC**
1. Echo time	0°	6.2 ± 0.3	13.4 ± 0.6	11.6 ± 0.2	5.0 ± 0.2
2. Noise	0°	11.2 ± 0.5	18.4 ± 0.7	15.9 ± 0.3	8.0 ± 0.5
3. Radius	0°	5.5 ± 0.3	10.5 ± 0.4	13.4 ± 0.2	3.6 ± 0.2
1. Echo time	90°	7.1 ± 0.3	6.4 ± 0.3	15.0 ± 0.1	5.6 ± 0.3
2. Noise	90°	9.8 ± 0.4	17.7 ± 0.9	15.6 ± 0.2	6.2 ± 0.2
3. Radius	90°	6.5 ± 0.3	7.8 ± 0.4	14.6 ± 0.2	4.5 ± 0.2

*The ground truth OEF was 22%. All values are the difference in OEF (%) from the ground truth (Equation 10)*.

**Figure 4:**
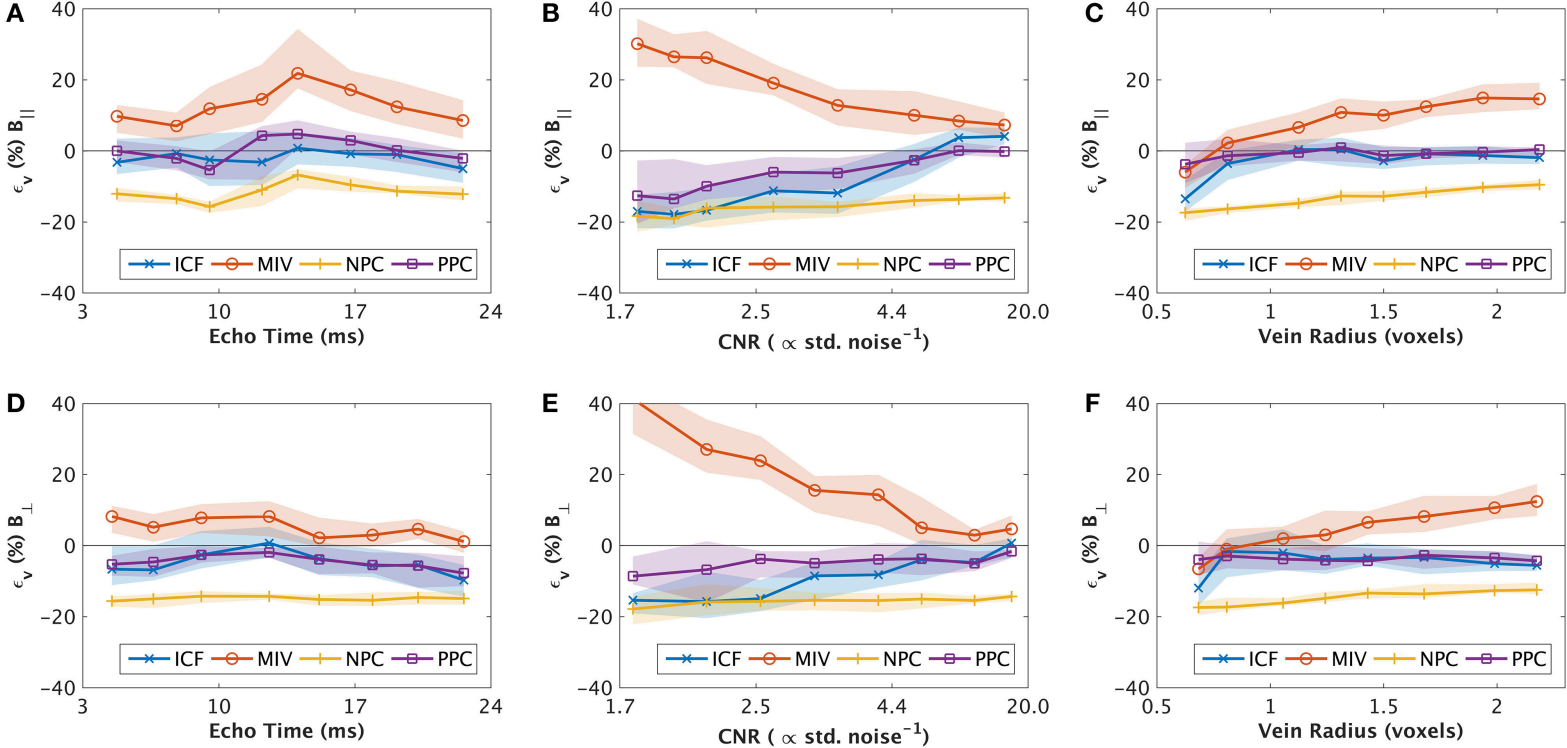
**Binned results of synthetic experiments 1, 2 and 3 for varied echo time (A,D) contrast-to-noise (B,E) and radius (C,E) respectively, showing the median (line) and inter-quartile range (shaded)**. Results are separated into those that were in parallel alignment to the main field (**A–C**, top row), and perpendicular to the main field (**D–F**, bottom row). The values are interpolated between estimates for each bin. The y-axis (ϵ_*v*_) shows the signed error in estimates of oxygen extraction fraction (OEF). Negative values are under-estimates of OEF. Unless stated in Table 2, all parameter values are described in Table 1.

The ICF error was lower than the NPC error in all experimental conditions, and lower than the MIV error in 5/6 experimental conditions. The MIV method had lower mean error in the remaining experiment (simulations of varied echo time with a perpendicular magnetic field), particularly at longer echo times (Figure 4D).

The median OEF error (and inter-quartile range) as a function of varied parameters is displayed in Figure 4 for each method. The ICF approach error generally tracked the PPC behavior as a function of the varied parameters. In cases of higher CNR (CNR ≥ 3) and in radius experiments with parallel magnetic fields, the PPC and ICF methods had near-zero bias (<5% OEF, Figures 4B,C,E). Notable exceptions (where the magnitude of PPC and ICF bias increased) included small vein radius (radius < 0.75 voxels, Figures 4C,F), low CNR (CNR <3, Figures 4B,E), and in cases of high CNR for ICF (CNR > 10, Figure 4B), which exhibited a positive bias. In all other cases, the ICF and PPC methods showed a small negative bias, which is indicative of OEF under-estimation.

The MIV approach had a consistent positive bias, whereas the NPC technique has a severe negative bias. The MIV over-estimation was exacerbated by larger vein radius (radius > 1.25 voxels, Figures 4C,F) and lower CNR (CNR <5, Figures 4B,E). The ICF approach demonstrated low error (lower than the MIV and NPV approaches) that was comparable to the PPC method (the “ideal” method) in all cases where vein radius was larger than 0.75 voxels and CNR was higher than 3.

ICF computation time was less than 3 s per slice. Twelve maps failed to converge prior to reaching the termination condition (from the total of 1,800 simulated maps). These 12 cases were included in the results above as the value of ϵ_fit_ in these cases was similar to the average value for all simulations.

### Systematic error investigation

The results of the systematic error investigation are presented in Table 4. The PPC method had the lowest systematic error. The MIV method in all simulations had a positive bias, despite the absence of noise. The ICF method under-estimated the vein radius in four of the six conditions (by up to 12.4%) and incurred a corresponding over-estimation of vein magnetic susceptibility in these cases. The systematic error experiments had a higher CNR than the images simulated in Figures 4B,E, which is expected with no added noise. The Figures 4B,E results, extrapolated to a higher CNR, are consistent with the systematic error results.

**Table 4:** **Examination of systematic error in noise-free simulations**.

**Vein center position (x,y)**	**Orientation of magnetic field**	**PPC (ϵ_*v*_)**	**PPC (ppm)**	**MIV (ϵ_*v*_)**	**MIV (ppm)**	**ICF (ϵ_*v*_)**	**ICF (ppm)**	**ICF (ϵ_*R*_)**	**ICF ($\hat{R}$)**
(0,0)	0°	1.2%	0.32	1.8%	0.32	1.2%	0.32	0.6%	1.31
(0.25, 0.25)	0°	0.7%	0.31	6.8%	0.39	5.5%	0.37	−10.8%	1.16
(0.5, 0.5)	0°	3.4%	0.35	7.9%	0.41	6.9%	0.39	−12.4%	1.14
(0,0)	90°	0.1%	0.30	0.7%	0.31	−0.1%	0.30	1.4%	1.32
(0.25, 0.25)	90°	1.3%	0.32	7.5%	0.40	5.9%	0.38	−9.4%	1.18
(0.5, 0.5)	90°	4.2%	0.36	8.9%	0.42	6.9%	0.39	−11.5%	1.15

*Exact QSM measurements are denoted ppm (ppm = χ_vein_−χ_background_). The vein center position is relative to the corner of the voxel [e.g., at (0.5, 0.5) the center of the voxel is aligned with the center of the vein]. All values are reported as signed values, i.e., negative values are under-estimates of the true value. The ground truth OEF was 22%, χ_vein_−χ_background_ was 0.30 and radius was 1.3 voxels*.

### *In vivo* results

**Figure 5:**
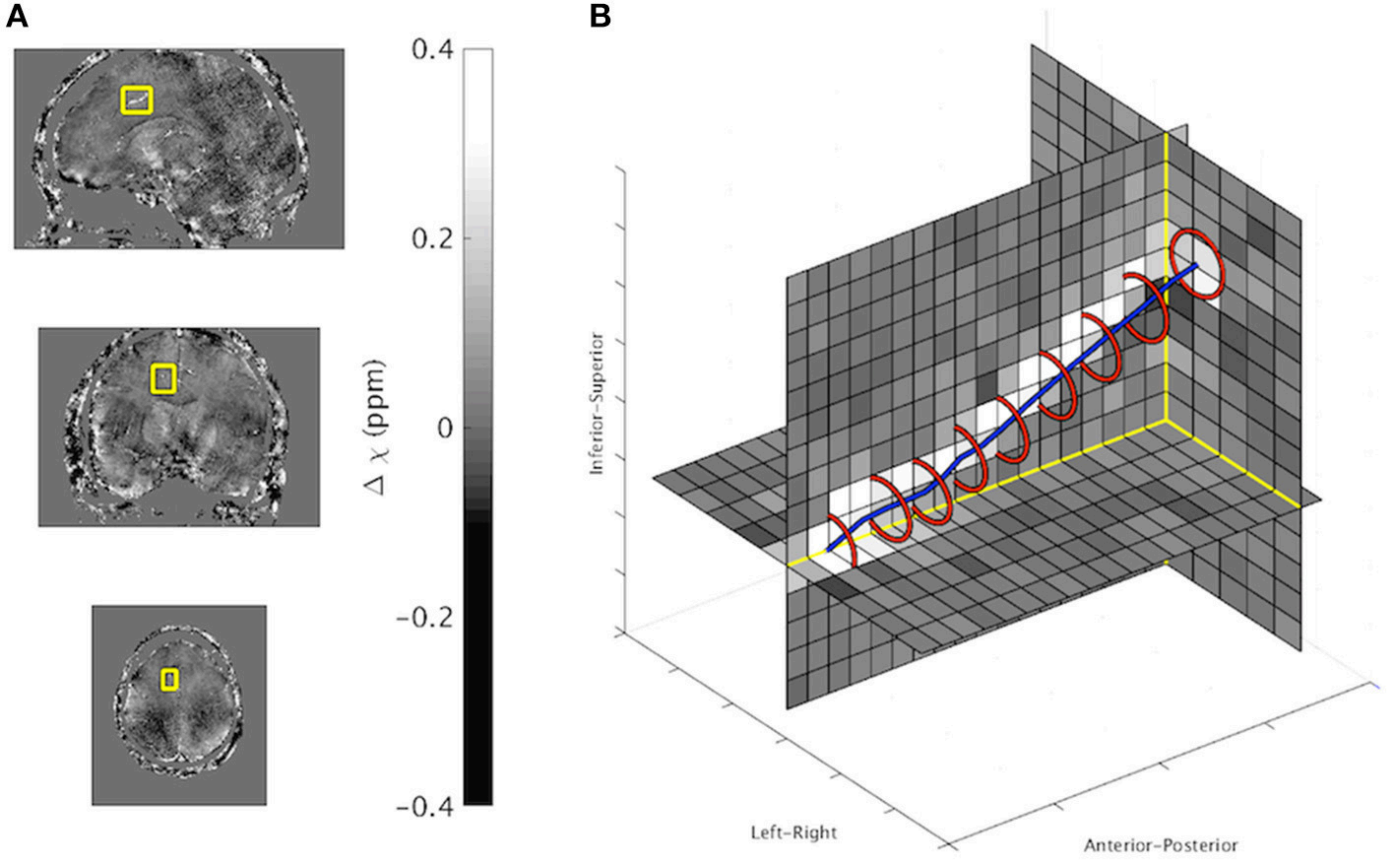
**(A)** *In vivo* QSM maps in sagittal (top), coronal (middle), and axial (bottom) orientation. The border of the vein analyzed is overlaid. **(B)** Zoomed in 3D display of vein region with overlaid ICF output (Red: Vein boundary, Blue: Line connecting vein center points). ICF estimates: χ_vein_ = 0.32 ppm, Radius 0.92. MIV estimate: χ_vein_ = 0.33 ppm.

An *in vivo* example output from the ICF method is presented in Figure 5. A full list of results for each *in vivo* image can be found in Table 5. ICF estimates of vein radius were between 0.63 and 1.59 voxels (0.38–0.96 mm). The difference between the MIV and the ICF estimates of χ_*vein*_ was found to correlate with estimated radius (Pearson's correlation coefficient *R* = 0.45, *p* < 10^−5^). This correlation was also demonstrated in the simulated data of the radius experiment (*R* = 0.39, *p* < 10^−5^).

**Table 5:** **Description of veins used for *in vivo* analysis with experiments results^a^**.

**Vein**	**Length (mm)**	**Angle from B0**	**Radius (mm)**	**Radius (voxels)**	**OEF**	
					**MIV**	**ICF**
Frontal cortical vein	6.6	5°	0.43 ± 0.05	0.71 ± 0.08	0.22 ± 0.03	0.23 ± 0.03
Frontal cortical vein	5.4	15°	0.50 ± 0.06	0.84 ± 0.10	0.20 ± 0.03	0.22 ± 0.06
Frontal cortical vein	7.2	20°	0.55 ± 0.08	0.91 ± 0.13	0.29 ± 0.07	0.28 ± 0.05
Frontal cortical vein	6.6	85°	0.58 ± 0.07	0.97 ± 0.12	0.25 ± 0.05	0.26 ± 0.05
Inferior cerebral vein	6.6	10°	0.58 ± 0.06	0.97 ± 0.09	0.23 ± 0.05	0.22 ± 0.05
Inferior cerebral vein	4.8	15°	0.63 ± 0.05	1.05 ± 0.09	0.31 ± 0.05	0.29 ± 0.05
Inferior frontal vein	6	80°	0.48 ± 0.05	0.80 ± 0.08	0.28 ± 0.02	0.25 ± 0.02
Inferior sagittal sinus	5.4	85°	0.61 ± 0.04	1.01 ± 0.07	0.26 ± 0.06	0.24 ± 0.06
Inferior sagittal sinus	6	85°	0.62 ± 0.06	1.03 ± 0.10	0.28 ± 0.06	0.24 ± 0.04
Inferior sagittal sinus	4.8	90°	0.70 ± 0.07	1.17 ± 0.11	0.26 ± 0.06	0.24 ± 0.05
Inferior sagittal sinus	6.6	90°	0.70 ± 0.08	1.17 ± 0.13	0.31 ± 0.04	0.28 ± 0.04
Inferior temporal vein	7.8	85°	0.44 ± 0.07	0.74 ± 0.12	0.29 ± 0.03	0.23 ± 0.03
Inferior temporal vein	4.8	85°	0.49 ± 0.08	0.81 ± 0.13	0.28 ± 0.03	0.29 ± 0.04
Inferior temporal vein	4.8	90°	0.51 ± 0.08	0.85 ± 0.13	0.18 ± 0.03	0.21 ± 0.03
Inferior temporal vein	4.2	35°	0.55 ± 0.06	0.91 ± 0.10	0.19 ± 0.04	0.16 ± 0.03
Internal cerebral vein	3.6	30°	0.96 ± 0.09	1.59 ± 0.15	0.21 ± 0.03	0.18 ± 0.02
Septal vein	4.8	50°	0.55 ± 0.08	0.92 ± 0.14	0.35 ± 0.01	0.25 ± 0.01
Septal vein	4.8	65°	0.56 ± 0.08	0.94 ± 0.14	0.35 ± 0.04	0.27 ± 0.02
Superior cerebral vein	7.2	5°	0.38 ± 0.05	0.63 ± 0.08	0.21 ± 0.05	0.18 ± 0.03
Superior cerebral vein	7.8	20°	0.49 ± 0.05	0.81 ± 0.09	0.20 ± 0.03	0.22 ± 0.03
Superior cerebral vein	6	85°	0.49 ± 0.08	0.81 ± 0.14	0.18 ± 0.04	0.18 ± 0.05
Superior cerebral vein	6.6	15°	0.51 ± 0.06	0.86 ± 0.10	0.27 ± 0.05	0.17 ± 0.05
Superior cerebral vein	7.2	15°	0.54 ± 0.09	0.89 ± 0.14	0.20 ± 0.04	0.22 ± 0.04
Superior cerebral vein	6	15°	0.58 ± 0.11	0.97 ± 0.18	0.22 ± 0.04	0.20 ± 0.05
Superior cerebral vein	4.8	10°	0.61 ± 0.12	1.01 ± 0.19	0.22 ± 0.05	0.20 ± 0.04
Superior cerebral vein	6.6	10°	0.64 ± 0.07	1.06 ± 0.11	0.23 ± 0.05	0.23 ± 0.05
Superior cerebral vein (trib.)	4.2	80°	0.52 ± 0.04	0.87 ± 0.07	0.42 ± 0.06	0.39 ± 0.05
Superior cerebral vein (trib.)	4.2	85°	0.54 ± 0.05	0.89 ± 0.08	0.18 ± 0.03	0.18 ± 0.03
Tentorial vein	7.2	90°	0.63 ± 0.03	1.05 ± 0.04	0.23 ± 0.04	0.22 ± 0.04
Vein of the cingulate sulcus	6	90°	0.52 ± 0.05	0.86 ± 0.08	0.44 ± 0.08	0.40 ± 0.06
Average	5.8	50°	0.56 ± 0.10	0.94 ± 0.17	0.35 ± 0.09	0.32 ± 0.08

a *Trib., denotes a tributary to the named vein*.

## Discussion

The ICF technique provided OEF estimates with lower mean error compared to the MIV and NPC method on synthetic QSM maps. The improved performance of the ICF method can be attributed to the inclusion of information in boundary voxels using a simple two-component linear partial volume model. Four key factors affecting imaging of small structures in QSM were examined: echo time, noise, image resolution and orientation with respect to the main magnetic field.

The close alignment between ICF behavior and PPC behavior, as a function of parameter values, is indicative of low partial volume errors and the accuracy of the ICF approach to estimating vein geometry. However, for smaller veins (*R* < 0.75 voxels) and low signal to noise (CNR < 3) the ICF error increases more rapidly than the PPC approach, suggesting a compounding of error from QSM reconstruction with the inability of ICF to accurately estimate the underlying geometry.

The sensitivity of the MIV method to noise is expected as it uses a single voxel value (maxima) that may be an outlier. In noise and radius simulations, the MIV error in OEF rapidly increased as noise and radius increased. The NPC method depicted a sustained and significant under-estimation of OEF, and a mean error of similar magnitude to the MIV approach in most cases. This under-estimation is expected when taking the mean of small veins where partial volume effects are substantial.

As discussed in the introduction more sophisticated models, such as a Gaussian mixture model, are unsuited as there are too few purely venous voxels to estimate model parameters from. By considering partial volume the ICF method allows more voxels to be included in the analysis, thereby decreasing sensitivity to noise. Additionally, all intermediate steps of the ICF method, such as column and row summations, employ all available voxels yielding robustness to noise in intermediate steps.

The radius experiments show the ICF method outperforming MIV for *R* > 0.75 voxels. The ICF method requires two grid intersections for accurate analysis (*x*_1_ and *x*_2_ in Figure 2). Depending on the vein center position relative to the image grid, there may only be one intersection for veins of less than 2 voxels in diameter. A large vein-tilt angle makes this case less likely (due to the elongated elliptical vein cross-section) and decreases the minimum vein size that can be analyzed with the ICF technique. However, when only one intersection occurs the area in one or both segments approaches zero (shaded regions in Figure 2, green and blue columns in Figure 1). In these cases the boundary of the vein is estimated to be touching but not crossing the grid line. As shown in the results for veins between 0.75 and 1.0 voxels in radius, this is a satisfactory minimum bound to provide some amount of useful partial volume correction (as no voxel can be entirely vein without two or more intersections occurring). A contra-assumption occurs for the MIV method, which assumes at least one voxel is entirely vein (and that it has a negligible amount of noise).

Similar results were obtained with both parallel and perpendicular magnetic field orientations. A sustained under-estimation of magnetic susceptibility (by the PPC method) was found when the magnetic field was perpendicular to the vein orientation. This effect was most pronounced in vein radius experiments and indicates an orientation bias in the QSM reconstruction.

In studies which examined larger draining veins, and brain regions using PET ^15^O, changes in OEF on the order of 10–20% have been found in many conditions including mild traumatic brain injury, multiple-sclerosis, tumor and stroke (Leenders et al., 1985; Sobesky et al., 2005; Ge et al., 2012; Doshi et al., 2015). Few studies examine veins other than the major sinuses, however a recent study excluded these large vessels when examining OEF in the elderly using QSM images (Ward et al., 2016b). In that study, the veins of interest were penetrating veins, such as the cortical pial vessels (typically 1–3 voxels in diameter), that were spatially localized to different cortical regions. A maximum intensity approach to partial volume correction was employed and significant OEF variations were identified between cortical brain regions on the scale of 10–20%. These studies may benefit from improved partial volume correction, with ICF and other methods, given the error observed in simulations of the maximum approach in this manuscript.

The reliability of ICF in *in vivo* veins as small as 0.63 voxels (0.38 mm) in radius was shown with consistent susceptibility estimates across slices and the replication of the characteristic patterns with MIV from the simulated data with respect to radius. MIV estimated lower susceptibility values in small veins and higher susceptibility in large veins. This pattern of bias was also observed in the simulation experiments.

Echo time experiments explored higher phase accumulation (longer echo times). All four methods examined had similar characteristic curves following the expected behavior of phase accumulation and partial volume. As the phase difference inside and outside the cylinder approached π, increased signal cancelation occurred due to destructive interference. This signal loss is in addition to $T_{2}^{*}$ decay, both of which contribute to a reduced signal-to-noise ratio. The MIV method error under these conditions demonstrates a high sensitivity to noise. This is reiterated in the noise experiments where the error of the MIV method increased much faster than the ICF method as the amount of noise added increased.

The PPC method error as a function of echo time, particularly where the magnetic field was parallel to the vein orientation, depicts the effects of phase accumulation and phase decoherence in QSM reconstruction without any partial volume artifacts in the analysis. Intra- and extravascular phase destructively interfere at an echo time of 11–12 ms. At these echo times, the signal-to-noise ratio is reduced due to signal cancelation, which may impact on QSM reconstruction. Similar echo time dependent errors have been identified in other imaging studies that focused on voxel constituents (Sood et al., 2016).

In this work, the ICF method takes a QSM map as an input and therefore results may be affected by susceptibility errors due to the QSM reconstruction itself. Variance was observed in the results from the iLSQR simulations, which may be attributable to the random number sequence used in the sampling, the integration of geometry, and/or the solvers within the iLSQR algorithm. The choice of QSM algorithm and the reconstruction parameters affect the accuracy of the QSM estimates. The systematic error in this study is indicative of this, with non-zero PPC error and a positive bias in maximum voxel intensities in the absence of noise. The systematic error was sensitive to the partial volume profile, i.e., the vein center position within the voxel. These findings suggest residual errors in the QSM reconstruction from the ill-posed nature of the dipole kernel inversion, and from the phase information lost in the complex sum of voxel constituents.

The dependence of MR gradient echo phase images on vessel (or cylinder) orientation to the main field also affects the accuracy of QSM maps due to the differing intra- and extravascular field contributions, and resultant signal-to-noise properties (Li et al., 2012; Fan et al., 2014). A residual bias has been reported in numerical simulations of QSM that was orientation dependent (Fan et al., 2014). This bias was possibly related to small values in k-space in the dipole kernel.

Techniques have been proposed to preserve high frequency components and address orientation-specific biases by incorporating structural priors into QSM reconstruction (Tang et al., 2013; Cetin et al., 2016). Improving the QSM algorithm that is used to pre-process the data before application of ICF is outside the scope of this paper; however it is noted that the geometry estimates provided by ICF may be useful in iterative QSM reconstruction techniques that employ image domain priors, such as Tang et al. (2013) and Cetin et al. (2016).

The ICF technique is applicable to cylindrical geometries in any image where partial volume effects are approximately linear (Equation 1). In this work ICF was applied to QSM images, as such χ was used in many of the equations, however it could be replaced with “flow,” “proton density” or in the general case “signal.” ICF may prove useful for analyzing both arterial and venous vessels in different body regions and modalities (other than QSM), as well as other applications where cylindrical geometries are present. As such, other suitable vessel-based applications include CT angiography, spin-echo imaging and phase contrast in arteries for flow velocity. There are also cylindrical image processing applications outside of medical imaging that may also benefit from the technique.

A limitation of ICF is the requirement that the vein intersects two grid lines in each dimension. This is not guaranteed to occur for veins smaller than one voxel in radius, which restricts the smallest veins suitable for ICF. This may be partially mitigated by vein position and tilt angle, as an elliptical cross-section has a higher in-slice radius than the vein itself. For particularly small veins, the contrast is unlikely to be sufficient to initially identify the veins. For larger veins, where more than 2 intersections are possible, it is expected that the centermost grid lines will provide the best estimates in Equation (4) due to the even distribution of area between the segments used.

The lack of ground truth for *in vivo* data inhibits measurements of method accuracy. Although consistency between cross-sectional slices of vein radius and intensity can be used as an indicator of accuracy, an ideal validation would include a physical phantom with known and complex vein geometry, realistic magnetic susceptibility distributions and thin inter-compartmental barriers. Alternatively, images could be acquired at a very high-resolution (e.g., 0.3 mm isotropic) and downsampled to simulate different levels of partial volume. The veins on the original images should experience fewer partial volume effects and could be used as a ground truth. An alternative approach to this problem could be to estimate vein geometry from *in vivo* data, and compare the acquired data with images simulated from these ICF estimates.

Normal aging and other neurological disease processes affect vessel size and metabolism in the brain, and ICF may aid in characterizing and distinguishing these two effects. Demonstration of its clinical utility requires a larger population dataset and application of ICF *in vivo* at lower field strengths that are more widely available in the clinic. We expect ICF to benefit vessel quantification from images acquired at 3T, with appropriate echo times for this field strength.

The ICF technique employs PPC to improve the accuracy of vein magnetic susceptibility estimates, and subsequent OEF measurements. Compared to the MIV and No Partial volume Correction (NPC) approaches, results from synthetic data demonstrated that ICF provides more accurate estimates of partial volume and venous OEF in the presence of noise and with varying vessel radius and echo time. Application of ICF in quantitation of vessel structure and physiology, such as OEF, will improve the accuracy and reliability of measurements.

## Author contributions

PW originally conceived of the method. AN, AF, PR, and PW contributed to the data acquisition. All authors (AF, AN, DD, DB, GE, PR, and PW) contributed to the analysis and interpretation of the data. Drafting was lead by PW and all authors (AF, AN, DB, DD, GE, PR) contributed to the critical revision and final presentation of the work.

### Conflict of interest statement

The authors declare that the research was conducted in the absence of any commercial or financial relationships that could be construed as a potential conflict of interest. The reviewer RP declared a shared affiliation, though no other collaboration, with one of the authors AF to the handling Editor, who ensured that the process nevertheless met the standards of a fair and objective review.
