# Supplementary material for: Improved Quantification of Cerebral Vein Oxygenation Using Partial Volume Correction
Source: Front Neurosci. 2017 Feb 27;11:89. doi: 10.3389/fnins.2017.00089 (PMC5326785; doi:10.3389/fnins.2017.00089)
Supplement: Supplementary file 1 [file AppendixA.docx]

# Appendix A

*Theorem A: The position and radius of a cylinder is uniquely identifiable by the fractional area partitioned by two non-parallel pairs of parallel chords on a cross-sectional plane given the cylinder orientation relative to the plane and the location of the chords.*

To prove Theorem A it is useful to begin with the case of a cross-sectional plane perpendicular to the cylinder axis giving a circular cross-section (Figure 2B). Only one set of parallel chords ($x_{1}$ and $x_{2}$) is depicted, and the below equations are for the x-axis.

The area (segment) partitioned by each chord is shaded in Figure 2C, and labeled $c_{1}$ and $c_{2}$ in Figure 2B. This area can be calculated using the area of a circle sector (Figure 2B $a_{1}$ and $a_{2}$), and a triangle from the chord intersections and circle center ($P=(p_{x},p_{y})$) (Figure 2B $b_{1}$ and $b_{2}$).

| $\text{Are}\text{a}_{\text{segment}}=\text{Are}\text{a}_{\text{sector}}-\text{Are}\text{a}_{\text{triangle}}$  $\text{Are}\text{a}_{\text{segment}}= \frac{R^{2}\theta}{2}-R^{2}\sin\frac{\theta}{2}\cos\frac{\theta}{2}=\frac{R^{2}\theta}{2}-\frac{R^{2}\sin\theta}{2}$ | Eq. 14 |
| --- | --- |

where $R$ is the radius of the circle. Dividing by the circle area ($\text{Area}_{\text{Total}}=\pi R^{2}$) removes the radius dependence on the right hand side and provides an expression for the segment fractional Area ($A= \frac{\text{Area}_{\text{segment}}}{\text{Area}_{\text{Total}}}$).

| $A=\frac{1}{2\pi}(\theta-\sin\theta)$ | Eq. 15 |
| --- | --- |

The circle center relative to the chords can be calculated with a right-angle triangle.

| $p_{x}-x_{1}=R\cdot\cos\frac{\theta_{1}}{2}$ | Eq. 16 |
| --- | --- |

The sum of this distance for two parallel chords is equivalent to the distance between the chords.

| $p_{x}-x_{1}+x_{2}-p_{x} =R\cdot\cos\frac{\theta_{1}}{2} +R\cdot\cos\frac{\theta_{2}}{2}$ | Eq. 17 |
| --- | --- |

Rearranging gives an expression for the radius.

| $R= \frac{x_{2}-x_{1}}{\cos\frac{\theta_{1}}{2}+\cos\frac{\theta_{2}}{2}}$ | Eq. 18 |
| --- | --- |

Taking the ratio of Eq. 16 and Eq. 17 and re-arranging gives an expression for the center position.

| $p_{x}=x_{1}+(x_{2}-x_{1})\frac{\cos\frac{\theta_{1}}{2}}{\cos\frac{\theta_{1}}{2}+\cos\frac{\theta_{2}}{2}}$ | Eq. 19 |
| --- | --- |

These equations are written in terms of the x-axis, and can be repeated for the y-axis to provide the full coordinates of the circle center ($p_{x},p_{y}$).

Extending this to a non-perpendicular cross-sectional plane with an elliptical shape requires transforming the axis dimensions by the major axis of the ellipse. The circular radius R is projected onto these axes to give $R_{x}$ and $R_{y}$. This is an affine transformation, which has the property that ratios of lengths and areas are preserved. Since all of the equations (excluding Eq. 18) are ratios of area or length, no modification is required. The radius calculation (Eq. 18) from each set of parallel chords must be transformed, as they are not relative measurements. This is performed differently for radii calculated using grid lines in the x-dimension,

| $R=\frac{R_{x}}{\sqrt{\left( \frac{\sin\phi}{\cos\varphi} \right)^{2}+\cos^{2} \phi}}$ | Eq. 20 |
| --- | --- |

and the y-dimension,

| $R=\frac{R_{y}}{\sqrt{\left( \frac{\cos\phi}{\cos\varphi} \right)^{2}+\sin^{2} \phi}},$ | Eq. 21 |
| --- | --- |

where $\phi$ is the angle of the ellipse’s major axis relative to the x-axis in the x-y plane, and $\varphi$ is the angle between the cylinder axis and the x-y plane (Figure 2A).

By taking Eq. 18, Eq. 19, Eq. 20, and Eq. 21 it is possible to calculate the center point and radius of a cylinder from a single cross sectional slice given the area of two segments in each dimension. In an imaging application, this area can be calculated from the pixel intensities directly if the elliptical cross-section spans three image columns in each dimension. An example is depicted in Figure 2B. In this case, summing the pixel intensities in the right hand column, and dividing by the total intensity of all pixels in the image will give the area of $c_{2}$. The same operation can be performed for pixels in the left column (left of x_1_) to complete the two area measurements for the x-axis ($c_{1}$ in Figure 2B). Repeating the process in the y-axis provides all the estimates required to calculate the above quantities. This requires zero contribution from the background to the pixel intensity, a property that is addressed in the Method section of the paper.
